# Ascorbic Acid Alleviates Damage from Heat Stress in the Photosystem II of Tall Fescue in Both the Photochemical and Thermal Phases

**DOI:** 10.3389/fpls.2017.01373

**Published:** 2017-08-08

**Authors:** Ke Chen, Minna Zhang, Huihui Zhu, Meiyu Huang, Qing Zhu, Diyong Tang, Xiaole Han, Jinlin Li, Jie Sun, Jinmin Fu

**Affiliations:** ^1^College of Resources and Environmental Science, South-Central University for Nationalities Wuhan, China; ^2^Key Laboratory of Plant Germplasm Enhancement and Specialty Agriculture, Wuhan Botanical Garden, The Chinese Academy of Sciences Wuhan, China; ^3^Key Laboratory of Catalysis and Materials Science of the State Ethnic Affairs Commission & Ministry of Education, College of Resources and Environmental Science, South-Central University for Nationalities Wuhan, China; ^4^Wuhan Kaidi Electric Power Environmental Co., Ltd. Wuhan, China

**Keywords:** L-ascorbate, heat stress, tall fescue, photochemical phase, thermal phase

## Abstract

L-Ascorbate (Asc) plays important roles in plant development, hormone signaling, the cell cycle and cellular redox system, etc. The higher content of Asc in plant chloroplasts indicates its important role in the photosystem. The objective of this study was to study the roles of Asc in tall fescue leaves against heat stress. After a heat stress treatment, we observed a lower value of the maximum quantum yield for primary photochemistry (φ_Po_), which reflects the inhibited activity of the photochemical phase of photosystem II (PSII). Moreover, we observed a higher value of efficiency of electron transfer from Q_B_ to photosystem I acceptors (δ_R0_), which reflects elevated activity of the thermal phase of the photosystem of the tall fescue. The addition of Asc facilitate the behavior of the photochemical phase of the PSII by lowering the ROS content as well as that of the alternative electron donor to provide electron to the tyrosine residue of the D1 protein. Additionally, exogenous Asc reduces the activity of the thermal phase of the photosystem, which could contribute to the limitation of energy input into the photosystem in tall fescue against heat stress. Synthesis of the Asc increased under heat stress treatment. However, under heat stress this regulation does not occur at the transcription level and requires further study.

## Introduction

Grasslands occupy about twice the land area of grain crops throughout the world (Mian et al., 2008) and play important ecological roles in the biosphere. For example, grasslands influence the carbon cycle, as well as the nitrogen cycle. Tall fescue (*Festuca arundinacea Schreb*) grows in temperate regions of the world, which is a major cool season forage and turf grass species (Chen et al., 2013; Wang and Huang, 2017). Additionally, it has been widely reported to be a model species for the rhizoremediation of various organic contaminants because of its well-developed root system and broad adaptability to different climates and environments (Lu and Zhang, 2014). Although tall fescue has a relatively higher thermal tolerance than other cool season turfgrasses, heat stress is still a major abiotic stress that limits its use across the world. Heat stress limits the growth and development of tall fescue in transitional and warm climatic regions, especially in summer, when high temperature can constrain growth, reduce turf quality, induce leaf withering and inhibit photosynthesis (Sun et al., 2015).

The photosynthetic activity of the chloroplasts is considered as the most heat sensitive cell function (Essemine et al., 2011). The linear electron transport chain in thylakoid membrane starts with the splitting of water and ends with the reduction of NADP^+^. Chlorophyll *a* fluorescence, which is measured under saturating continuous light, is a highly sensitive, non-destructive, and reliable tool for studying the energy flow and photosynthetic efficiency in the photosystem (Stirbet and Govindjee, 2011). OJIP curves from chlorophyll *a* fluorescence studies can be generally divided to photochemical phase and thermal phase. The photochemical phase is mainly related to the reduction of quinone A (Q_A_), and the thermal phase follows the photochemical phase, which disappears at subfreezing temperatures (Stirbet, 2012). In particular, Photosystem II (PSII) oxidizes water through an oxygen evolution complex (OEC, namely, the water splitting complex), which contains five oxygen atoms in addition to four Mn atoms and one Ca atom, which form a Mn_4_CaO_5_-cluster (Umena et al., 2011) and produce high amount of reactive oxygen species (ROS) in plants (Pfannschmidt, 2003; Pospíšil, 2012). The ROS are responsible for heat-induced damage to PSII (Yamashita et al., 2008). The D1 protein in the reaction center (RC) in PSII accepts the electron from the splitting of water by OEC and transfers it to plastoquinone molecules (PQ) through photochemical reactions, and the heat stress induces the cleavage of the DE-loop of the D1 protein.

L-Ascorbate (Asc) has crucial roles in plants, although its major plant synthesis pathway, the L-galactose pathway, has only been confirmed in the past decade (Smirnoff and Wheeler, 2000; Linster and Clarke, 2008; Smirnoff, 2011). It is one of the most abundant low molecular weight antioxidants in plants, which also play important roles in plant development, hormone signaling, the cell cycle and expansion, the cellular redox system and cofactors for enzymes (Debolt et al., 2007; Foyer and Noctor, 2011; Mellidou et al., 2012). In leaves, the concentration of Asc is approximately 1–5 mM, whereas the content of Asc in chloroplast is approximately 25 mM, which suggests that it may play an important role in photosynthesis and transmembrane electron transport (Wheeler et al., 1998). Under heat stress, the OEC is damaged due to the removal of extrinsic proteins and the release of calcium and manganese ions from their binding sites (Barra et al., 2005), which results in the blocking of electron transport from OEC to the Try_z_^+^ (D1 protein, Tyr161). Moreover, it has been showed that under heat stress, Asc, as a large pool of alternative electron donors, could donate electrons to PSII to sustain electron transport activities in leaves (Toth et al., 2009, 2011).

In this study, we aimed to determine whether Asc could alleviate heat stress induced damage to tall fescue PSII. To this purpose, exogenous addition of Asc was conducted to study the role of Asc in tall fescue leaves against heat stress. Chlorophyll *a* fluorescence measurements were employed to study both the effects of Asc on the photosynthetic apparatus as well as the productivity of photosynthetic organisms. Additionally, thylakoid membranes of the tall fescue leaves were extracted to verify the ability of the Asc to serve as an alternative electron donor. Additionally, regulation of Asc synthesis under heat stress was studied and discussed to increase our understanding of the role of Asc against abiotic stress.

## Materials and Methods

### Plant Materials and Growth Conditions

Tall fescue commercial type ‘Huntdog 5’ was seeded in pots with sand as growth medium (Luo et al., 2011). After germination, plants were kept in a greenhouse (day/night temperature: 24/20°C) with a 16 h photoperiod (light intensity: 300 μmol photons m^-2^ s^-1^) for 50 days to allow the tall fescue roots and shoots to be established (Fu and Huang, 2001). The seedlings were fertilized twice a week with 50 mL of half-strength Hoagland’s solution.

### Reagent and Heat Stress Treatment

Reagent treatments for the leaves were performed by a vacuum infiltration method since it supports high efficiency transport of reagents into plant leaves (Filippou et al., 2012). In this study, 20 mM sodium ascorbate (Sigma, St. Louis, MO, United States) was used for exogenous addition of ascorbic acid (marked as “Asc” in the figures and tables) (Uchida et al., 2002). Briefly, the third fully expanded leaves were cut from a petiole and immersed in 1/2 strength Hoagland’s solution alone (marked as “Ct” in the figures and tables) or with Asc in a 15-cm petri dish. Then, the petri dishes were moved into a desiccator for vacuum infiltration for 15 min in the dark (Ederli et al., 2006; Attallah et al., 2007).

Heat stress treatments of tall fescue leaves were conducted using the same methods as our previous research (Chen et al., 2013). Briefly, after the reagent treatments, the petioles of the leaves were immersed in 1 cm of 1/2 strength Hoagland’s solution and maintained in a falcon tube with Asc or no Asc. Then, the tubes were placed in a growth cabinet with a temperature of 24 ^o^C (labeled “24” in the figures and tables) or 44°C (labeled “44” in the figures and tables), and a light intensity of 100 μmol photons m^-2^ s^-1^, and humidity of 80% for the indicated durations. After the treatments, the leaves were subjected to variety analysis.

### Chlorophyll *a* Fluorescence Transient

In our study, all measurements related to Chl *a* fluorescence were measured by a PAM chlorophyll fluorometer (PAM 2500, Heinz Walz GmbH) and the measurements were repeated at least five times. After a 25-min dark adaptation period, OJIP transients were induced by red light (650 nm) with a 3000 μmol photons m^-2^s^-1^ light intensity and recorded by the instrument. The OJIP transient data were analyzed using the JIP-test (Chen et al., 2013). The indexes of φ_Po_ (maximum quantum yield for primary photochemistry, namely *F*_V_/*F*_M_), Ψ_E0_ (efficiency/probability with which a PSII trapped electron is transferred from Q_A_ to Q_B_), δ_R0_ (efficiency/probability with which an electron from Q_B_ is transferred to PSI acceptors), ABS/RC (absorbed photon flux per RC), γ_RC_ (probability that a PSII Chl molecule functions as a RC), PI_ABS_ (performance index for energy conservation from photons absorbed by PSII antenna, for the reduction of Q_B_) and PI_Total_ (performance index for energy conservation from photons absorbed by PSII antenna until reduction of PSI acceptors) were employed in to study the energy flow and photosynthetic activities of tall fescue leaves.

### Preparation of Thylakoid Membranes

The preparation of thylakoid membranes refers to the methods mentioned by Robinson (Robinson and Yocum, 1980) with slight modification. Briefly, 30 g of tall fescue leaves was crushed within 50 mL of precooled 0.4 M NaCl, 2 mM MgCl_2_, 0.2% bovine serum albumin (BSA), and 20 mM Tricine (pH 8.0) solution by a Waring blender. The homogenate was then filtered through four layers of cheesecloth, and centrifuged at 4°C, and 500 g for 3 min. The supernatant was then centrifuged at 4°C, and 4000 *g* for 10 min to collect broken chloroplasts. The pellets were resuspended in 10 mL of precooled 0.1 M NaCl,5 mM MgCl_2_, 0.2% BSA, and 20 mM Tricine (pH 8.0) solution, and centrifuged at 4°C, and 4000 *g* for 10 min twice; the pellet was then washed with the solution mentioned above. Finally, thylakoid membranes were suspended in precooled 0.4 M sucrose, 15 mM NaCl, 0.2% BSA, and 20 mM Tricine (pH 8.0) solution. The thylakoid concentration was adjusted to 200 μg Chl/mL before subsequent treatment.

### Regeneration of the K-Step of Tall Fescue Thylakoid Membranes

Tall fescue thylakoids were incubated with water (Ct) or 20 mM ASC (Asc) in a 44°C water bath for 1 min to inactivate OEC. Then, 20 μL of thylakoids (200 μg Chl/mL) was dropped onto a nitrocellulose membrane, and after 25 min of dark adaption, Chl *a* fluorescence transients were induced and recorded by two 5-ms saturated pulses that were spaced 200 ms apart.

### Determination of ASC

Tall fescue leaves were crushed in liquid nitrogen, and 0.05-g samples were added to 1 mL of perchloric acid, shaken vigorously, and centrifuged at 4°C, and 14000 *g* for 15 min. Then, 400 μL of supernatant was mixed with 65 μL of a 0.1 M HEPES-KOH (pH = 7) solution, and 98 μL of 1.7 M K_2_CO_3_ was added to adjust the pH to 4–5. The mixture was centrifuged at 4°C, and 14000 *g* for 15 min, and 50 μL of supernatant was mixed with 940 μL of 60 mM phosphate buffer (pH = 6.3). Then, 1 unit of ascorbate peroxidase (APX, Sigma, St. Louis, MO, United States) was added to the above mixture, and the absorbance at 265 nm after 2 minutes was recorded. The content of ASC was calculated by use of a standard curve.

### Determination of Hydrogen Peroxide and Superoxide Radical

The hydrogen peroxide and superoxide radical contents were measured by histological staining method (Ramel et al., 2009). Tall fescue leaves were immersed with 1 mg mL^-1^ DAB (3,3′-diamino-benzidine, Sigma, St. Louis, MO, United States) staining solution (hydrochloric acid, pH 3.8) for 30 min. Stained leaves were then bleached in an acetic acid–glycerol–ethanol (1/1/3) (v/v/v) solution at 100°C for 5 min, and stored in a glycerol–ethanol (1/4) (v/v) solution for 30 min. Next, the leaves were ground in liquid nitrogen, homogenized in 0.2 M HClO_4_, and centrifuged for 10 min at 12000 *g*. A450 was immediately measured and compared with a standard curve containing known amounts of H_2_O_2_ in a 0.2 M HClO_4_-DAB solution.

In the case of superoxide radical (O_2_^•-^) determination, leaves were immersed and infiltrated with 2 mg mL^-1^ nitroblue tetrazolium (NBT, Sigma, St. Louis, MO, United States) staining solution (25 mM KOH-HEPES buffer, pH 7.8) and incubated in the dark for 30 min. The stained leaves were bleached in an acetic acid-glycerol-ethanol (1/1/3) (v/v/v) solution at 100°C for 5 min and stored in a glycerol–ethanol (1/4) (v/v) solution for 30 min. Then, the NBT-stained leaves were ground in liquid nitrogen, and the formazan content in the obtained powder was solubilized in 2 M KOH-DMSO (1/1.16) (v/v), and centrifuged for 10 min at 12000 *g*. The A630 of the solution was immediately measured and compared with a standard curve obtained from known amounts of NBT.

### APX Activities

Tall fescue leaves were crushed in liquid nitrogen, and crude enzymes were extracted by 50 mM phosphate buffer (pH = 7.8). After centrifuging at 4°C, and 12000 *g* for 15 min, the supernatant was used to determine the APX activity following the method introduced by Nakano (Nakano and Asada, 1981). Briefly, the reaction solution contained 50 mM acetate (pH = 5.8), 5 mM H_2_O_2_, 10 mM Asc, and 3 μM EDTA. After the addition of the enzyme supernatant, the absorbance at 290 nm was recorded for 2 min with 10 s intervals, and 1 unit of APX activity was defined as the decrease of 0.01 absorbance per minute.

### Quantitative RT-PCR Analysis

Total RNA was isolated and purified from treated leaves according to the protocol of RNeasy^®^ plant mini kits (Qiagen). The expressions of selected genes were analyzed by real-time quantitative reverse transcriptase (RT-PCR) using the fluorescent intercalating dye SYBRGreen with a detection system (Opticon 2, MJ Research, Waltham, MA, United States). The *VTC2* coding mRNA sequence was amplified with the following primers: forward (5′-ACCTTCACAGGAGGATGCTG-3′) and reverse (5′-CCACGCGGAACTCTGTAGGG-3′). The *VTC4* coding mRNA sequence was amplified with the following primers: forward (5′-GACCACAAGTTCATCGGCGAG-3′) and reverse (5′-CCACGAACAGCTGTGAAAAGCT-3′) (Hu et al., 2014). *eEF1A* (s) was used as a standard control for RT-PCR. The two-step RT-PCR procedure was performed following the methods we described earlier.

### Statistical Analysis

Each experiment was repeated at least five times. The values in the figures or tables are provided as the mean ± SD. Statistical analyses were performed by Duncan’s multiple range tests or two-way ANOVA as indicated in the figures or tables. Different letters in the figures or tables indicate statistically significant differences at *P* < 0.05.

## Results

### Effects of Asc on Tall Fescue OJIP Transients against Heat Stress

**FIGURE 1:**
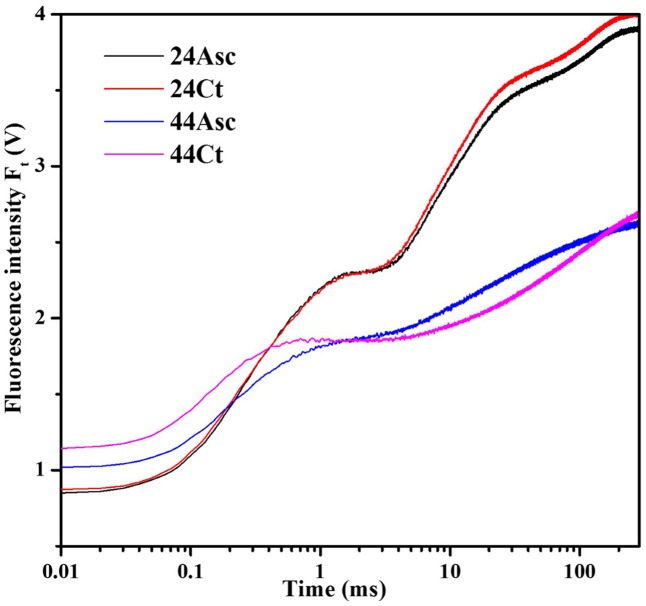
Effects of heat stress and L-Ascorbate (Asc) on OJIP fluorescence transients in tall fescue leaves. After the reagent treatments, the petiole of the leaves was immersed 1 cm deep within distilled water kept in a falcon tube with Asc (24Asc and 44Asc) or not (24Ct and 44Ct). Then the tubes were placed in a growth cabinet with the temperature of 24°C (24Ct and 24Asc) or 44°C (44Ct and 44Asc), and light intensity of 100 μmol photons m^-2^ s^-1^, and a humidity of 80% for 4 h. Then after a 25-min dark-adaption period at 24°C, the OJIP curves were induced by a red light provided by a PAM 2500. Chl *a* fluorescence emissions were recorded by the instrument and the most typical curves are shown here.

**FIGURE 2:**
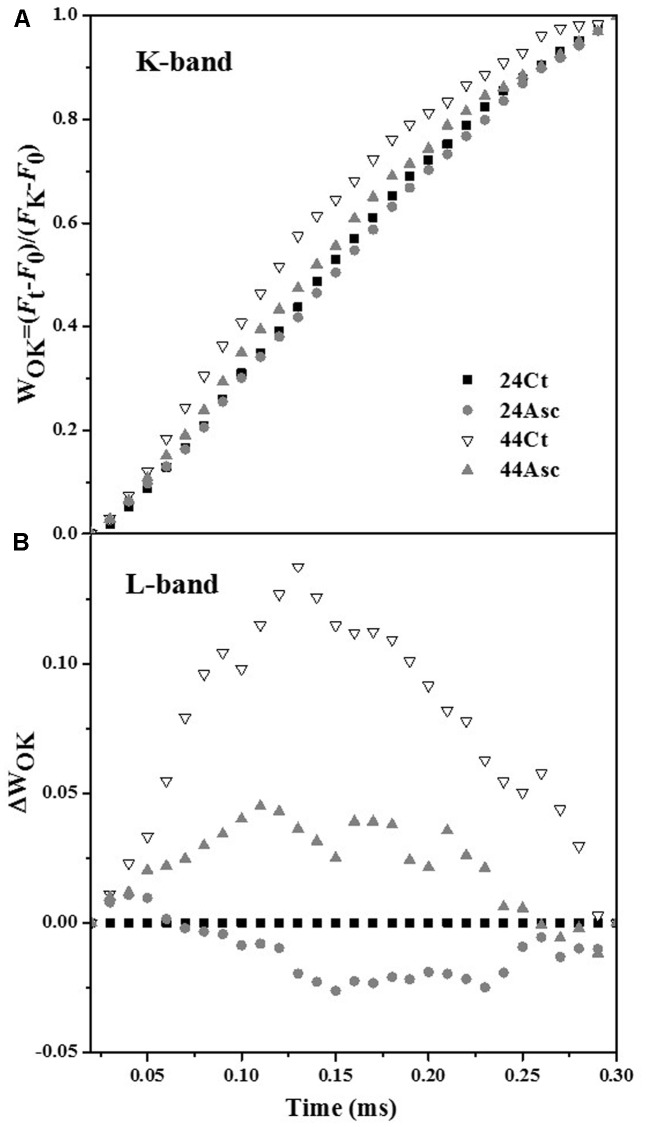
Effects of heat stress and Asc on the K-band and L-band in tall fescue leaves. The differences in the heat stress and Asc treatment samples (**Δ**V_t_), **(A)** between *F*_0_ and *F*_K_: W_OK_ = (*F*_t_–*F*_0_)/(*F*_K_–*F*_0_) and **(B)** the differences of the heat stress samples to the control sample (24Ct) (ΔW_OK_).

Tall fescue leaves were subjected to heat stress and Asc treatment to study the role of Asc in PSII against heat stress using the OJIP fluorescence transient method. The fluorescence transient curves are shown in **Figure 1**. From the figure, heat stress treatment significantly affected the OJIP fluorescence transient curves. After heat stress, higher initial fluorescence as well as lower maximum fluorescence were observed. Additionally, the clear K step of heat-stress treated samples was observed at 0.3 ms, which indicates thermal damage to the PSII. Additionally, compared 44Ct curve, the 44Asc curve showed a lower K step, which indicates that Asc treatment alleviated the damage from heat stress. To better understand the effect of heat stress and Asc treatment on the K step, K-band, and L-band of OJIP curves were studied and are shown in **Figure 2**. **Figures 2A,B** depict the relative variable fluorescence between *F*_0_ and *F*_K_ (W_OK_) as well as the differences of transients in samples treated with heat stress and Asc minus the room temperature transient (24Ct) (**Δ**W_OK_). In **Figure 2B**, the sequence from upper to lower was 44Ct, 44Asc, 24Ct, and 24Asc, which indicates heat stress damage on PSII and the positive role of Asc in this process.

The basic fluorescence parameters were extracted from the curves and are provided in **Table 1**. The JIP-test was used to further understand the energy flow and electron transport in PSII in this study, and the relevant data are provided in **Table 2**. Compared to 24Ct, 44Ct had lower values of φ_Po_, γ_RC_ and PI_ABS_ as well as higher values of δ_R0_ and ABS/RC. Additionally, compared to 44Ct, 44Asc had lower values of Ψ_E0_, δ_R0_, ABS/RC, and PI_Total_ as well as higher values of φ_Po_, γ_RC_, and PI_ABS_.

**Table 1:** Basic photosynthetic parameters extracted from the OJIP transient curves.

Parameters	24Ct	24Asc	44Ct	44Asc
*F*_0_	0.88 ± 0.10	0.86 ± 0.10	1.16 ± 0.14	1.03 ± 0.13
*F*_300_	1.65 ± 0.21	1.65 ± 0.18	1.74 ± 0.23	1.56 ± 0.2
*F*_J_	2.29 ± 0.32	2.31 ± 0.29	1.85 ± 0.24	1.86 ± 0.24
*F*_I_	3.56 ± 0.45	3.46 ± 0.44	2.14 ± 0.28	2.3 ± 0.31
*F*_M_	4.07 ± 0.54	3.94 ± 0.51	2.72 ± 0.31	2.66 ± 0.35

*^∗^F_0_: Minimal reliable recorded fluorescence at 20 μs; *F*_300_: fluorescence value at 300 μs; *F*_J_: fluorescence value at the J-step (2 ms) of OJIP; *F*_I_: fluorescence value at the I-step (30 ms) of OJIP; *F*_M_: fluorescence value at the peak of OJIP test; Values shown are the means ± SD (*n* = 5)*.

**Table 2:** Photosynthetic parameters deduced by the JIP test analysis of fluorescence transients.

Parameters	24Ct	24Asc	44Ct	44Asc
φ _Po_	0.784 ± 0.12 a	0.782 ± 0.13 a	0.574 ± 0.09 c	0.614 ± 0.08 b
Ψ_E0_	0.557 ± 0.08 a	0.530 ± 0.07 a	0.554 ± 0.08 a	0.487 ± 0.06 b
δ_R0_	0.290 ± 0.04 c	0.291 ± 0.04 c	0.666 ± 0.11 a	0.448 ± 0.06 b
ABS/RC	2.783 ± 0.42 c	2.787 ± 0.40 c	5.835 ± 0.88 a	4.152 ± 0.61 b
γ_RC_	0.264 ± 0.03 a	0.264 ± 0.04 a	0.146 ± 0.02 c	0.194 ± 0.03 b
PI_ABS_	1.641 ± 0.27 a	1.449 ± 0.25 a	0.287 ± 0.04c	0.363 ± 0.05 b
PI_Total_	0.671 ± 0.11 a	0.596 ± 0.19 a	0.573 ± 0.10 a	0.295 ± 0.06 b

*Subscript “0” indicates that the parameter refers to the onset of illumination. Values are given as the means ± SD (*n* = 5), and different letters indicate statistically significant differences at *P* < 0.05*.

### Effect of Heat Stress and Asc Treatments on ROS Production in Tall Fescue Leaves

**FIGURE 3:**
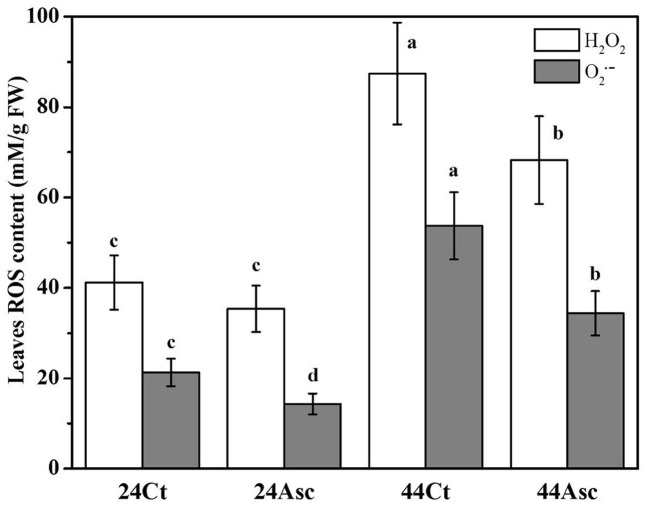
Effect of heat stress and Asc on ROS production in tall fescue leaves. Values are given as the means ± SD (*n* = 5), and two-way ANOVA was used to study the differences between heat stress and Asc treatments. Different letters indicate statistically significant differences at *P* < 0.05.

To better understand the role of Asc in tall fescue under heat stress, the ROS content of tall fescue leaves subjected to the heat stress and Asc treatment were studied in **Figure 3**. From the figure, compared to 24Ct, the additional 20 mM Asc treatment (24Asc) decreased the content of H_2_O_2_ and O_2_^•-^ of tall fescue leaves by approximately 16 and 48%, respectively. The heat stress treatment (44Ct) increased the content of H_2_O_2_ and O_2_^•-^ of leaves by approximately 2.1- and 2.5 -fold than those of tall fescue under room temperature (24Ct), respectively. After addition of 20 mM Asc (44Asc), the content of H_2_O_2_ and O_2_^•-^ of tall fescue leaves under heat stress decreased by approximately 28 and 56%, respectively.

### Alternative Electron Donor Studies of Tall Fescue Thylakoids

**FIGURE 4:**
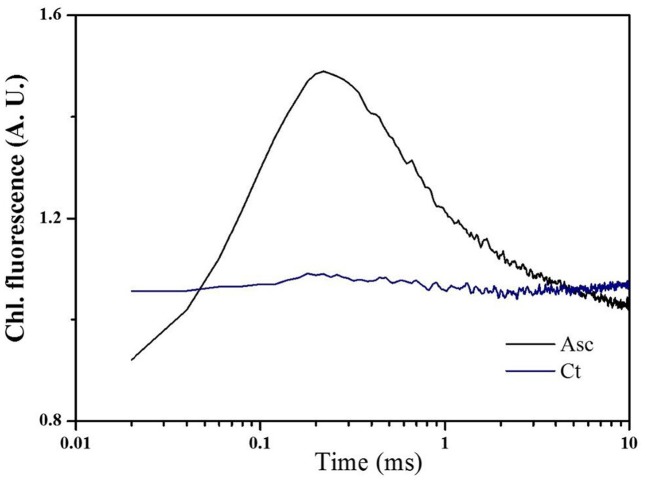
Effects of Asc on the electron donor side of PSII under heat stress. Tall fescue thylakoids were incubated with water (Ct) or 20 mM Asc (Asc) in a 44°C water bath for 1 min to inactivate the oxygen evolution complex. Then, 20 μL of thylakoids (200 μg Chl/mL) was dropped onto a nitrocellulose membrane, and after a 20-min dark adaption period, Chl a fluorescence transients were induced and recorded by two 5-ms saturated pulses that were spaced 200 ms apart.

To better understand alternative electron donor of tall fescue PSII, tall fescue thylakoids were extracted and incubated at 44°C for 1 min to inactivate OEC, and Asc was used to study exogenous electron donors in PSII (**Figure 4**). From the figure, regeneration of the K-step as a function of the dark interval between two 5-ms saturated pulses was studied. Compared to the control (Ct), an additional 20 mM Asc treatment (Asc) peaked the Chl *a* fluorescence transient curves at 0.3 ms.

### Effects of the Heat Stress Treatment on the APX Activities, Asc Contents, and Transcription Levels of VTC4 and VTC2

**FIGURE 5:**
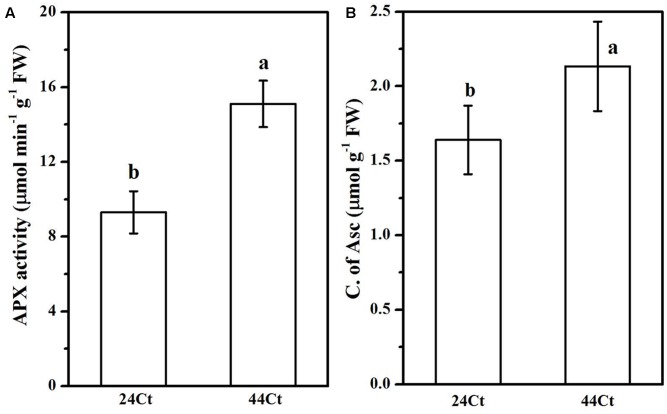
Effects of heat stress on the APX activity **(A)** and production of ASC **(B)**. Tall fescue leaves were vacuum infiltrated with water (24Ct, 44Ct) for 15 min in the dark. Then, they were moved into a growth cabinet with a temperature of 24 or 44°C, a light intensity of 100 μmol photons m^-2^ s^-1^ and a humidity of 80% for 4 h. Afterward, tall fescue leaves were ground in liquid nitrogen and used to determine the concentration of Asc as well as APX activity. Values are provided as the means ± SD (*n* = 5), and different letters indicate statistically significant difference at *P* < 0.05 among the treatments according to Duncan’s multiple range tests.

**FIGURE 6:**
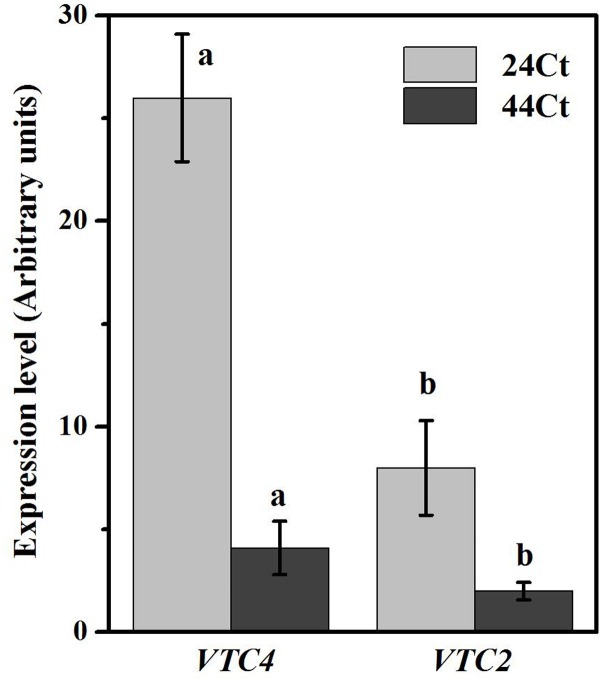
Effects of heat stress on the Asc-related gene expression. Tall fescue leaves were vacuum infiltrated with water (24Ct, 44Ct) for 15 min in the dark. Then, they were moved into a growth cabinet with a temperature of 24 or 44°C, a light intensity of 100 μmol photons m^-2^ s^-1^ and a humidity of 80% for 4 h. Afterward, tall fescue leaves were ground in liquid nitrogen and used to study mRNA transcription. Values are given as the means ± SD (*n* = 5), and different letters indicate statistically significant difference at *P* < 0.05 among the treatments from Duncan’s multiple range tests.

To further understand the regulation of Asc synthesis for tall fescue leaves against heat stress, the APX activity and Asc content were studied and are shown in **Figure 5**. In **Figure 5**, after heat stress, the tall fescue APX activity (44Ct) and Asc content (44Ct) increased significantly. Additionally, the expression profiles of *VTC2* and *VTC4* were studied, and the data are shown in **Figure 6**. After the heat stress treatment (44Ct), the transcription levels of *VTC2* and *VTC4* significantly decreased.

## Discussion

Even though the tall fescue has considerable heat tolerance, heat stress limits its growth and development in transitional and warm climatic regions (Sun et al., 2015). In this study, the effects of exogenous Asc on the tall fescue against heat stress and regulation of Asc synthesis of tall fescue under heat stress have been studied. On the electron donor side, heat stress treatment increased initial fluorescence (F_0_) of tall fescue leaves, as shown in **Figure 1**. After dark adaption, all of the primary quinone acceptors in PSII are in an oxidized state, and F_0_ is the level of fluorescence emission of PSII induced by weak illumination. F_0_ refers to the concept of open, close and inactivation of the RC in PSII (Tsimilli-Michael and Strasser, 2008). As shown in **Table 2**, heat stress treatment significantly decreased the value of γ_RC_ (44Ct versus 24Ct), which indicates that heat stress treatment inactivated the RC in PSII. This response may be related to the aggregation of Light Harvest Complex II (Tang et al., 2007) or increase in the Q_B_ non-reducing center (Mehta et al., 2010). The K-step (0.3 ms) observed in 44Ct means that after heat stress, the connection between RC and OEC decreased, and electron transport from OEC to the tyrosine residue (Try_z_^+^, D1-Tyr161) was inhibited. **Figure 2** explores the K-band and L-band of the OJIP curves of tall fescue leaves under room temperature and heat stress. Heat stress treatment significantly increased the amplitude of the L-band (44Ct versus 24Ct), indicating that after heat stress treatment, the PSII units of tall fescue leaves were less grouped or that less energy was exchanged between independent PSII units, namely, there was a lose of stability and an increase in fragility.

In this study, the JIP test was used to study the energy flow of PSII of tall fescue leaves subjected to heat stress treatment. The JIP test evaluated the three major steps that occurred in electron transport in photosystem, namely, trapping photons, electron transport after Q_A_^-^ to intersystem electron acceptors and reduction of end acceptors (Stirbet and Govindjee, 2011), as well as the energy flow efficiency of these three steps, which was reflected through the values of φ_Po_, Ψ_E0_, and δ_R0_. After heat stress treatment, we observed a lower value of φ_Po_ and a higher value of δ_R0_, and in this study, there was no significant difference between the values of Ψ_E_ under room temperature and heat stress treatment. Thus, heat stress treatment inhibited the activities of PSII in the photochemical phase of the OJIP curves and the activity of PSII in the thermal phase of the OJIP curves was elevated.

Asc is a multifunctional metabolite in plants and plays an important role in plants against abiotic stresses. In this research, the multiple roles of Asc in tall fescue against heat stress were studied. Additionally, 20 mM Asc was used to incubate tall fescue leaves before heat stress. Addition of Asc facilitated the behaviors of the photosystem. In the photochemical phase, the addition of Asc reduced the amplitude of the K-band at room temperature (**Figure 2A**, 24Ct versus 24Asc) and under heat stress (**Figure 2A**, 44Ct versus 44Asc). Because the amplitude of the K-band corresponds to electron transport from OEC to the Try_z_^+^ (Tsimilli-Michael and Strasser, 2008), the addition of Asc facilitated electron transport to Try_z_^+^.

That may because, firstly, Asc could scavenge ROS, both enzymatically and nonenzymatically in the chloroplast stromas (Tsimilli-Michael and Strasser, 2008). High ROS produced in a water-oxidation reaction are extremely destructive to the D1 protein, and heat stress treatment could enhance the susceptibility of this photosynthetic apparatus (Takahashi et al., 2004). The addition of 20 mM Asc significantly decreased the level of ROS in tall fescue leaves under room temperature as well as heat stress (**Figure 3**), which alleviated the toxicity of ROS to the D1 protein and improved the behavior of electron transport from OEC to Try_z_^+^. The decreased ROS content in 44Asc may have also contributed to the recovery of RC in PSII, as shown in the increased value of γ_RC_ in **Table 2** (44Asc versus 44Ct).

Secondly, when the activity of OEC was inhibited under heat stress treatment, Asc could acted as an alternative electron donor of water to maintain the electron transport chain in PSII (Toth et al., 2009, 2011). In this study the role of Asc as an alternative electron donor of tall fescue thylakoids was verified in **Figure 4** through regeneration of the K step of the thylakoids after two 5-ms short saturating pulses separated by 200 ms dark durations. The treatment of thylakoids at 44°C for 1 min would inactive the function of OEC of splitting water as well as providing electron to Try_z_^+^. In this case, the first 5 ms short saturating pulse would consume electrons donated by Tyr_z_ and form Try_z_^+^, and rereduction of Tyr_z_^+^ after 200 ms was inhibited due to the impairment of OEC as well as lose of alternative electron donor in thylakoid prepartion procedure (Toth et al., 2009). Thus the second 5 ms short saturating pulse (at 200 ms, after the first 5 ms short saturating pulse) could not induce signal as shown in Ct in **Figure 4**. However, the addition of Asc induce a clear peak at 0.3 ms, which suggests that in tall fescue Asc could serve as an alternative electron donor to provide electrons to PSII via Try_z_^+^ (Srivastava et al., 1997; Toth et al., 2011).

It has been widely accepted that under heat stress, the photochemical phase of the photosystem is inhibited due to inactivation of RC or damage to OEC, and so on. Due to the roles of Asc in regulating ROS and serving as an alternative electron donor in PSII, the performance of the photochemical phase of tall fescue treated with Asc should be largely elevated, which was demonstrated by the increased values of φ_Po_ and γ_RC_ of tall fescue leaves pretreated with 20 mM Asc before heat stress treatment (44Asc versus 44Ct, **Table 2**). Additionally, we observed that, under heat stress, after pretreatment with Asc, the value of δ_R0_ decreased by approximately 48.6%, which indicated that there was lower activity of the thermal phase in the photosystem. Havaux (1996) reported that the PSI of potato leaves could catalyze an electron flow from reductants against heat-stress inhibited PSII activities, and the elevated activities of the thermal phase of the photosystem may be an important strategy for plants to regulate their energy flow in the photosystem to respond to heat stress. However, our previous studies showed that the heat tolerant tall fescue genotype under heat stress treatment had a relatively lower value of δ_R0_ (Chen et al., 2014) compared to the heat sensitive genotype, which correlates well with the results obtained in this study. Thus, in tall fescue, the increased activity of PSII in the thermal phase of the OJIP curves may be harmful, and the lower activity of the PSII may contribute to limiting the energy input into the photosystem as well as limiting the production of ROS to resist the heat stress. This result was also in line with our findings, in which pretreatment with Asc significantly decreased the values of PI_Total_ under heat stress (**Table 2**).

Regulation of APX activity and Asc synthesis of tall fescue under the heat stress treatment were also studied. Heat stress significantly increased the APX activity and Asc content in the tall fescue leaves. We observed that the transcription level of *VTC4* and *VTC2* decreased after the heat stress treatment. The *VTC2* gene is the last missing enzyme in the L-galactose pathway in Asc synthesis in higher plants (Linster and Clarke, 2008), and *VTC2* encods GDP-L-galactose phosphorylase, which is the first committed step in Asc synthesis. The transcription level of *VTC2* in Arabidopsis decreases with supplementation of Asc, which suggests that there is feedback inhibition in Asc synthesis in plants (Dowdle et al., 2007). Additionally, heat stress decreases *VTC2* transcripts in kiwifruit leaves (Li et al., 2013). The *VTC4* encoded L-galactose 1-phosphate phosphatase (GPP) is a bifunctional enzyme that affects myoinositol and Asc synthesis in plants (Torabinejad et al., 2009), and its expression level is reduced after exposure to heat stress in tomato leaves. These findings agree with the results we obtained in this study and suggest that in tall fescue, heat stress induces the synthesis of Asc, which inhibits genes transcription of the Asc synthesis pathway through feedback control. Thus, the regulation of elevated Asc after heat stress may occur at other levels, for example, histone modification and phosphorylation of target enzymes, which requires further study.

## Conclusion

In this study, heat stress treatment inhibited the activity of the photochemical phase of PSII and elevated the activity of the thermal phase of the photosystem of tall fescue. The addition of Asc facilitated the behavior of the photochemical phase of the PSII by reducing the ROS content as well as acting as an alternative electron donor to provide electron to the tyrosine residue of the D1 protein. Additionally, exogenous Asc reduced the activities of the thermal phase of the photosystem, which contributed to limiting the energy input in the photosystem of tall fescue against heat stress. Synthesis of Asc increased under heat stress. However, this regulation did not occur at the transcription level and requires our further study.

## Author Contributions

KC, MZ, HZ, and QZ wrote the main manuscript text; MH and DT prepared figures; XH and JL prepared the tables, JS and JF designed the overall of this study. All authors reviewed the manuscript.

## Conflict of Interest Statement

The authors declare that the research was conducted in the absence of any commercial or financial relationships that could be construed as a potential conflict of interest.
